# Innovative Experimental Ultrasound and US-Related Techniques Using the Murine Model in Pancreatic Ductal Adenocarcinoma: A Systematic Review

**DOI:** 10.3390/jcm12247677

**Published:** 2023-12-14

**Authors:** Andrea Coppola, Dario Grasso, Federico Fontana, Filippo Piacentino, Roberto Minici, Domenico Laganà, Anna Maria Ierardi, Gianpaolo Carrafiello, Fabio D’Angelo, Giulio Carcano, Massimo Venturini

**Affiliations:** 1 Diagnostic and Interventional Radiology Unit, Circolo Hospital, ASST Sette Laghi, 21100 Varese, Italymassimo.venturini@uninsubria.it (M.V.); 2 Department of Medicine and Technological Innovation, Insubria University, 21100 Varese, Italy; 3 Radiology Unit, Dulbecco University Hospital, 88100 Catanzaro, Italy; miniciroberto@gmail.com (R.M.);; 4 Department of Experimental and Clinical Medicine, Magna Graecia University of Catanzaro, 88100 Catanzaro, Italy; 5 Radiology Unit, IRCCS Ca Granda Ospedale Maggiore Policlinico, 20122 Milan, Italy; 6 Department of Medicine and Surgery, Insubria University, 21100 Varese, Italy; fabio.dangelo@uninsubria.it; 7 Orthopedic Surgery Unit, ASST Sette Laghi, 21100 Varese, Italy; 8 Emergency and Transplant Surgery Department, ASST Sette Laghi, 21100 Varese, Italy

**Keywords:** pancreatic cancer, experimental animal models, mouse, diagnostic imaging, ultrasound imaging, ultrasonic therapy, ultrasonography, interventional, high-intensity focused ultrasound ablation, translational research

## Abstract

Pancreatic ductal adenocarcinoma (PDAC) is a cancer with one of the highest mortality rates in the world. Several studies have been conductedusing preclinical experiments in mice to find new therapeutic strategies. Experimental ultrasound, in expert hands, is a safe, multifaceted, and relatively not-expensive device that helps researchers in several ways. In this systematic review, we propose a summary of the applications of ultrasonography in a preclinical mouse model of PDAC. Eighty-eight studies met our inclusion criteria. The included studies could be divided into seven main topics: ultrasound in pancreatic cancer diagnosis and progression (n: 21); dynamic contrast-enhanced ultrasound (DCE-US) (n: 5); microbubble ultra-sound-mediated drug delivery; focused ultrasound (n: 23); sonodynamic therapy (SDT) (n: 7); harmonic motion elastography (HME) and shear wave elastography (SWE) (n: 6); ultrasound-guided procedures (n: 9). In six cases, the articles fit into two or more sections. In conclusion, ultrasound can be a really useful, eclectic, and ductile tool in different diagnostic areas, not only regarding diagnosis but also in therapy, pharmacological and interventional treatment, and follow-up. All these multiple possibilities of use certainly represent a good starting point for the effective and wide use of murine ultrasonography in the study and comprehensive evaluation of pancreatic cancer.

## 1. Introduction

Pancreatic ductal adenocarcinoma (PDAC) is one of the most lethal tumours with a five-year survival rate inferior to 10% and a growing incidence;thus, it may soon become the second leading cause of cancer death [1,2]. It is characterized by a prominent desmoplastic stroma that limits therapeutic drug access and is highly resistant to standard chemo- and radiotherapy.

Recently, several studies have been conductedusing preclinical experiments for new therapeutic strategies in genetically engineered mouse models of pancreatic cancer [3,4,5,6,7].

New invitro models have been developed and improved in the last two decades, including tridimensional-culture spheroids and organoids. However, animal studies remain of fundamental importance before clinical studies are performed. Orthotopic grafting and syngeneic grafting are robust modelsused to test drug efficiency in a tumour and its microenvironment, and mice remain the most studied type of animal model in pancreas research [8].

In mice, the pancreas develops from two endodermal outgrowths of the primitive gut that fuse to give rise to the branched ductular mass characteristic of the exocrine gland. In humans, the gland is distinct and well-defined, whereas in mice it is a rather diffuse organ enclosed in the dorsal mesentery. The adult mouse pancreas is softer than the human one, and it is surrounded by the stomach, duodenum, proximal jejunum, and spleen [9,10]. Macroscopically, three lobes can be identified: the duodenal, the splenic (which is the largest), and the gastric. Respectively, they correspond to the head, the body/tail, and the pyramidal process of the human pancreas [11].

The use of animals for research purposes has long been a matter of debate in respect to animals’ sentience, the sufferance that might be caused to them during the experiments, and whether the justification for such harms is acceptable [12]. Today, studies on animals are based on the application of the Three Rs (replacement; reduction; refinement) and their tendency to respond to these concerns [13]. Especially regarding the second R, imaging techniques are of fundamental importance; if it is necessary to use animals, as few animals as possible should be used to achieve the study objectives. The main advantages of in vivo imaging techniques, as they do not require animal sacrifice, is the possibility of longitudinal studies, thus allowing a fewer number of animals and a lower cost to laboratories [9].

The ultrasound devices dedicated to the study of animal models, such as rodents, include high-frequency probes up to 60 MHz, through which high-resolution images can be obtained [9]. In addition to their diagnostic capabilities, ultrasound devices are also able to contribute, with a large number of therapeutic and interventional techniques to research. Experimental ultrasound, CEUS, and US-related techniques were widely employed using the murine model in different tumours to promote new drugs, molecules, or other innovative therapies with promising translational perspective [14,15,16,17,18,19,20,21,22,23].

The purpose of this systematic review was to analyse the role of ultrasound-based techniques in preclinical research for pancreatic cancer in the murine model.

## 2. Materials and Methods

A systematic literature search of major databases was conducted of murine studies investigating the diagnostic accuracy and utility of conventional ultrasound imaging for pancreatic adenocarcinoma in orthotopic mouse models. The study followed the Preferred Reporting Items for a Systematic Review and Meta-analysis of Diagnostic Test Accuracy (PRISMA) [24].

### 2.1. Search Strategy

A standardized search was performed in PubMed, using the following search terms: (“pancreatic tumor” and “ultrasound” and “murine”, “pancreatic tumor” and “ultrasound” and “mouse”, “pancreatic cancer” and “ultrasound” and “murine”, “pancreatic cancer” and “ultrasound” and “mouse”, “pancreatic adenocarcinoma” and “ultrasound” and “mouse”, “pancreatic adenocarcinoma” and “ultrasound” and “murine”). The search was conducted from February 2013 to May 2023 with no language restrictions.

### 2.2. Study Selection

Two authors screened and selected studies independently based on the eligibility criteria described below. Studies identified from different databases were de-duplicated after screening. Articles that passed the initial screening were reviewed for the full text.

### 2.3. Eligibility Criteria

Studies were considered eligible for this systematic review if they fulfilled the following criteria: (1) murine studies investigating the usefulness of ultrasound-based techniques for pancreatic cancer; (2) all diagnostic tests must have been performed on the mouse model of pancreatic adenocarcinoma; and (3) both prospective and retrospective studies were eligible.

We excluded studies when they met one of the following criteria: (1) experimentation on humans or animals other than mice; (2) studies based on investigation techniques different from ultrasounds, such as computed tomography and magnetic resonance imaging; and (3) cases with other types of abdominal tumours different from pancreatic cancer.

## 3. Results

**Figure 1:**
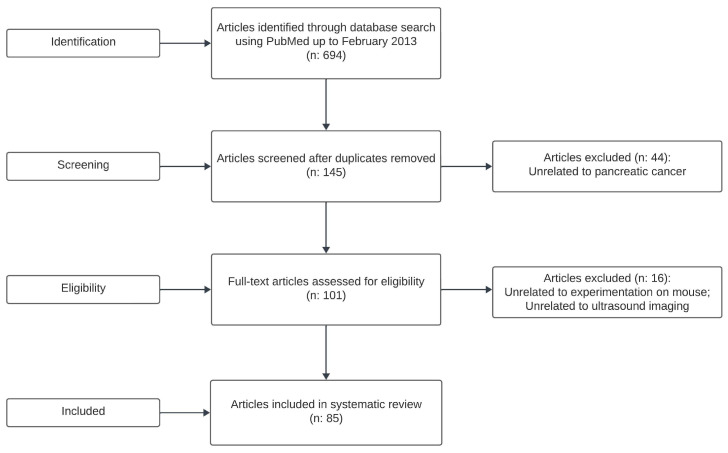
PRISMA flow diagram.

One hundred and forty-five studies were identified in our search. After assessing the titles and abstracts, 101 full texts were screened, as shown in Figure 1. Based on our selection criteria, 13 of those studies were excluded, while 88 studies met our inclusion criteria.

The included studies could be divided into seven main topics: ultrasound in pancreatic cancer diagnosis and progression (21 papers) [3,4,5,6,25,26,27,28,29,30,31,32,33,34,35,36,37,38,39,40,41]; dynamic contrast-enhanced ultrasound (DCE-US) (five papers) [6,42,43,44,45]; microbubble ultrasound-mediated drug delivery (18 papers) [7,46,47,48,49,50,51,52,53,54,55,56,57,58,59,60,61,62]; focused ultrasound (23 papers) [43,63,64,65,66,67,68,69,70,71,72,73,74,75,76,77,78,79,80,81,82,83,84]; sonodynamic therapy (SDT) (seven papers) [58,69,85,86,87,88,89]; harmonic motion elastography (HME) and shear wave elastography (SWE) (six papers) [67,68,90,91,92,93,94,95]; ultrasound-guided procedures (nine papers) [96,97,98,99,100,101,102,103,104].

In six cases, the articles fit into two or more sections [6,43,58,67,68,69].

## 4. Discussion

**Figure 2:**
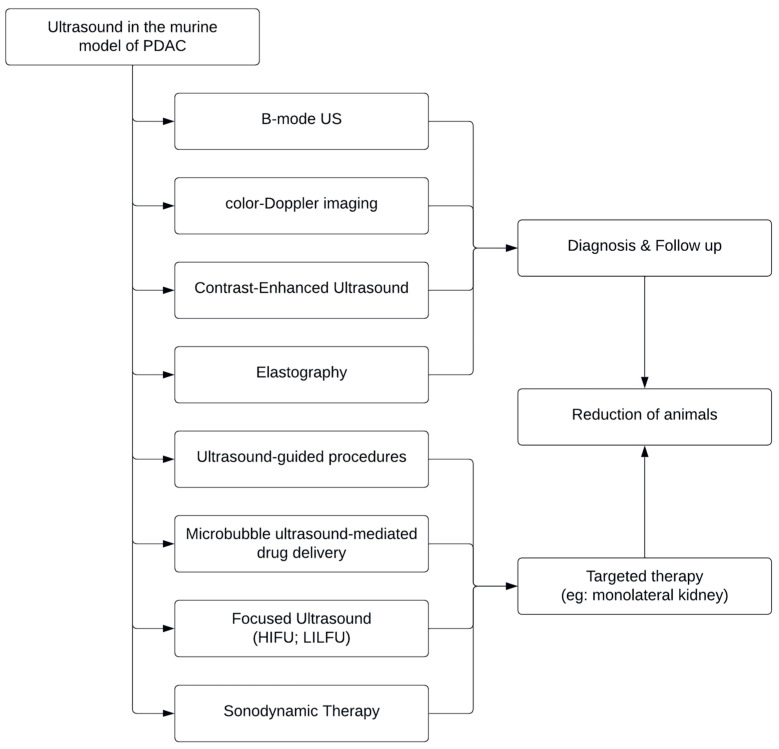
Applications of each type of ultrasound in murine models of pancreatic ductal adenocarcinoma (PDAC).

Although the role of ultrasound in the diagnosis and progression of pancreatic adenocarcinoma is certainly a broad field of application for this imaging technique, it has been shown that the majority of studies were concerned with the therapeutic potential of HIFU as an effective treatment for pancreatic cancer in mouse models. In addition, the combination of ultrasound and microbubble has previously shown particular interest as a tool used to enhance tissue distribution and intracellular drug delivery, such as chemotherapeutics and other anticancer drugs, as it resulted from our research (Figure 2).

### 4.1. Ultrasound in Pancreatic Cancer Diagnosis and Progression

Ultrasound in pancreatic disorders is a very useful imaging modality from screening to diagnosis. The main ultrasonographic features of pancreatic cancer are represented by its isoechogenicity or mild hypoechogenicity, irregularity of margins, and the presence of a central hyperechogenic region as the tumour enlarges. Other findings may include dilatation of the main pancreatic duct and its branches, atrophy, pancreatic cysts, dilatation of the bile duct, and lymphadenopathy [105].

Through imaging studies performed on transgenic mouse models, such as ultrasound, color Doppler imaging, or nonlinear contrast imaging frames, better early diagnosis and progress on therapeutic treatments could be achieved [6,25]. Hruban RH et al. have offered an update on pancreatic intraepithelial neoplasia (PanINs) as a precursor to invasive pancreatic cancer in genetically engineered mouse models [26]. In a recent study, Thy1 (thymocyte differentiation antigen 1) was identified and validated as a new biomarker in the diagnosis of pancreatic adenocarcinoma that can be outlined by ultrasounds in mice [27]. To define tumour-staging criteria using magnetic resonance (MR) and ultrasound (US), a four-class tumour staging system has been defined, ranking from stage 1 to 4 [28]. In vivo molecular imaging using Thy1-scFv (single-chain variable fragment) conjugated to an ultrasound contrast agent (MBThy1-scFv) demonstrated signal enhancement on a transgenic pancreatic ductal adenocarcinoma (PDAC) mouse model, suggesting potential for the early diagnosis of PDAC [29].

Endosonography with fine-needle aspiration biopsy (EUS-FNA) has become a widely available clinical tool used to diagnose numerous different benign and malignant lesions in humans. EUS-FNA is frequently used for tissue-based diagnoses, such as lymphatic diseases (ranging from tuberculosis/sarcoidosis to malignant lymphoma) or solid tumours (such as pancreatic carcinoma, neuroendocrine tumours, sub-epithelial gastrointestinal tumours, and others) [106]. Chiba M et al. used endoscopic ultrasound-guided fine needle aspiration (EUS-FNA) to quantify the S100P protein to discriminate between pancreatic adenocarcinoma (PCA) and benign pancreatic lesions (BPL). They found a significantly higher concentration of the S100P protein in the EUS-FNA samples rather than in the BPL cases [30]. EUS-FNA was also used to collect some samples of pancreatic ductal adenocarcinoma (PDAC) to test their responsiveness to treatments, such as for the dose of the 5-aza-dC DNA methyltransferase inhibitor [31]. Other studies have suggested a high correlation between fluorescence imaging and ultrasound imaging in assessing tumour burden and tumour progression in orthotopic mouse models of human cancer [32,33], while Rojas JD et al. demonstrated that combining BLI (bioluminescence imaging) and robotic US could provide an effective screening tool for pancreatic cancer in mouse models [34]. Several studies have analysed the role of high-resolution ultrasound in monitoring tumour onset, tumour volume quantification, tumour growth, metastatic progression, and therapeutic response in genetically engineered pancreatic cancer models [3,4,5,35,36,37,38,39]. Some of these cases have highlighted the relevance of ultrasound-guided photoacoustic imaging (US-PAI) providing multiparametric information on tumour vasculature and function and demonstrating the importance of changes in tissue oxygen saturation to predict treatment response, particularly tumour growth rate [40,41].

### 4.2. Dynamic Contrast-Enhanced Ultrasound (DCE-US)

Due to a low signal-to-noise ratio, Doppler US can outline blood flow information only in relatively large vessels, and it cannot evaluate microvasculature and tissue perfusion. By their physical properties, US contrast agents transcend this limitation. Their structure is an outshell of proteins, lipids, or polymers filled with air or various gases to form a microbubble. When a US contrast agent is administered, the backscatter of the ultrasound waves in the vascular system is enhanced by resonance within sonic windows. This results in the marked amplification of the signals from the blood flow and provides additional information about the microvasculature [107,108,109].

In this sense, the role of CEUS in pancreatic injury found on ultrasoundscould be very useful for making a more rapid diagnosis, especially of pancreatic cancer [110]. CEUS plays an important role in the differential diagnosis and characterization of pancreatic lesions thanks to the ability of the contrast agent to evaluate the haemodynamics of organs or lesions, as well as in the flow signal of arterial vessels [105].

Barrefelt A. et al. explored the potential application of air-filled polyvinyl alcohol microbubbles (PVA-MBs) as ultrasound contrast agents to visualize blood flow within the tumoral lesion in mouse models of pancreatic cancer. The authors also marked the bubbles with a near-infrared fluorophore to use them as a contrast agent for multimodal imaging with 3D-fluorescence imaging co-registered with 3D-μCT imaging [42]. DCE-US can be useful not only in the diagnostic phase but also for therapeutic monitoring. Kim JH et al. used DCE-US in PANC-1- nude mice to investigate therapeutic response after treatment with gemcitabine, high-intensity focused ultrasound (HIFU), and a combination of them [43]. Non-invasive microbubble contrast-enhanced ultrasound imaging offered a satisfying method for monitoring and quantifying vascular effects of antitumoral therapy, such as sunitinib or anti-vascular endothelial growth factor (VEGF) monoclonal antibodies, and/or gemcitabine [44,45].

To measure the kinetics of tumour blood flow (rise time, time to peak, and wash-in rate), Chen CT et al. injected a bolus of a microbubble contrast agent into the mice via tail vein catheterization and assessed the dynamics of the agent with ultrasound-based harmonic imaging [6].

### 4.3. Microbubble Ultrasound-Mediated Drug Delivery

With recent and significant advances in ultrasound and contrast agent technology, it has been possible to analyse the therapeutic ultrasound-mediated microbubble oscillation, which has demonstrated that this method can increase the permeability of the microvessel walls, improving the release and absorption of drugs into target tissues. To increase the permeability of microvessels, it is necessary to temporarily create pores in the cell membranes, and this is possible through a phenomenon called sonoporation, which is based on the use of high-intensity ultrasound and microbubbles or other cavitation agents [111]. Sonoporation succeeds optimally in improving drug absorption through the microbubble-potentiated enhancement of microvascular permeability [111]. Using low-intensity ultrasound, sonoporation can be caused by microbubbles oscillating with a stable motion, or stable cavitation, to stimulate the absorption of different drugs, while at higher ultrasound intensities, the explosive growth and collapse of microbubbles is achieved to induce the formation of pores and the direct cytoplasmic absorption of drugs [112].

The combination of ultrasound and microbubbles has offered promising results as a tool to increase cell membrane permeability and to obtain a better tissue distribution and intracellular drug release;thus, the therapeutic efficacy of the ultrasound-mediated drug delivery of molecules, nanoparticles, and other therapeutic agents (like nab-paclitaxel, gemcitabine, haemoglobin S) is evaluated in multiple preclinical studies [46,47,48,49,50,51,52]. Some of these use the mechanism of ultrasound-targeted microbubble destruction (UTMD) [53,54,55]. It has resulted in a viable approach for the targeted release of the drug to solid tumours and involves using low-intensity ultrasound to interrupt microbubbles in the tumour vasculature, releasing encapsulated or attached drugs, for example, using a combination of irinotecan and oxaliplatin [56], a combination of gemcitabine and paclitaxel [57], or a combination with chemo-sonodynamic therapy [58]. Feng S et al. explored the use of low-intensity ultrasound (LIUS) combined with microbubble to enhance tumour blood perfusion and improve local drug concentration in nude mice bearing pancreatic cancer [59]. In other works, a SonoVue^®^ ultrasound contrast agent was used in combination with a focused ultrasound transducer to produce sonoporation in the localised tumoral region only in an orthotopic xenograft model of human pancreatic cancer to demonstrate that combined sonoporation and gemcitabine therapy significantly impedes primary tumour development [60]. Moreover, Kotopoulis S et al. compared three different microbubble formulations to determine which of them was the best for low-intensity sonoporation of pancreatic ductal adenocarcinoma [61].

Locally focused ultrasound at a frequency of 1 MHz was employed to activate oxygen peroxide to deliver NO in pancreatic xenografted tumour-bearing nude mice to generate more highly reactive oxygen-contained species, demonstrating an effect in killing pancreatic tumour cells [66]. Kulkarni et al. used ultrasound and bubbles of echogenic polymersomes to target, penetrate, and deliver anticancer drugs in the hypoxic tissues of mice growing xenograft tumours of pancreatic cancer cells by subcutaneously injection [62].

### 4.4. Focused Ultrasound

HIFU (high-intensity focused ultrasound) is a non-invasive ultrasound tool based on focusing high-frequency ultrasound on a specific tissue to obtain a thermal effect and the subsequent tissue modulation up to percutaneously ablation [113]. The therapeutic potential of HIFU is represented by its ability to achieve the localized deposition of high-energy doses in deep tissues of the body, with the advantage of not damaging surrounding tissues and not making use of needles, probes, or electrodes, unlike other ablation methods [114]. This procedure has been evaluated for the treatment of both benign and malignant tumours, such as uterine fibroids in women [115], localized prostate cancer [116], neurological disorders [117,118,119], painful bone metastases [120], and various malignancies including in the liver, kidney, and breast [121,122,123]. One of the most common fields of application of HIFU is certainly human pancreatic tumours, in which HIFU ablation has an important role in palliative treatment thanks to its capacity to induce coagulative necrosis as well as tissue destruction and to reduce pain [124,125]. Pulsed high-intensity focused ultrasound (pHIFU) has been shown to be very useful as an effective treatment for pancreatic cancer in mouse models and, due to enhanced vascular permeability, in the disruption of tumour barriers and enhanced drug penetration into tumoral tissue through acoustic cavitation [63,64,65]. HIFU ablation was used to substantially restrict PDAC (pancreatic ductal adenocarcinoma) hematogenous metastasis and provided effective tumour control locally, as shown by Yu Q et al. [66]. In addition, HIFU treatment could be monitored by a new clinical tool, such as the harmonic motion imaging-guided focused ultrasound (HMIgFUS) technique, which offers the ability to successfully identify thermal injury and control lesion growth or reduction in real time in vitro and in vivo in abdominal tumours, such as pancreatic cancer [67,68]. Maeda M et al. showed that sonodynamic therapy, through the use of HIFU, produces cytotoxic reactive oxygen species (ROS) in and around cancerous cells in a mouse model of pancreatic cancer [69].

Several treatment strategies for pancreatic cancer have been designated and evaluated by combining focused ultrasound ablation, such as histotripsy, hyperthermia, electroporation, and antibody therapies, with conventional ablation techniques [70]. Some of these have demonstrated the feasibility of using histotripsy for pancreatic cancer ablation. Histotripsy is an ultrasound-based, non-invasive technique that, similar to other tumour ablation techniques, is able to mechanically destroy target cells. To study the effects of histotripsy on pancreatic adenocarcinoma, the release of potential antigens obtained by the histotripsy treatment of pancreatic adenocarcinoma in vitro model and by other ablation techniques was compared. Su JJ et al. evaluated irreversible electroporation (IRE) treatment in a nude mouse model, obtaining many potential advantages over conventional ablation techniques. In addition, hyperthermia induced by HIFU can be an efficient system used to enhance the localised release and spread of doxorubicin or gemcitabine inside pancreatic cancer to induce cell death and regions of apoptosis and necrosis [70,71,72,73,74].

Several papers showed that combining HIFU ablation with chemotherapy can contribute to improve survival. In fact, in many of these studies, HIFU was used to analyse the feasibility and efficacy of a combination of focused US and gemcitabine or doxorubicin in a mouse model of pancreatic cancer. This combined treatment was shown to bea more effective therapeutic response compared to other treatments [43,75,76,77].

In other cases, the synergistic effects of HIFU and the chemotherapic-loaded microbubble complex has been shown to increase the assimilation and therapeutic reaction of conventional chemotherapy in murine orthotopic pancreatic ductal adenocarcinoma and can effectively suppress tumour growth [78,79,80,81]. Additionally, the combined treatment of HIFU + immune checkpoint inhibitors was investigated in pancreatic cancer murine models, demonstrating apoptosis in pancreatic cancer cells [82,83].

Similar to HIFU, low-intensity low-frequency ultrasound (LILFU) may downregulate the expression levels of ABC transporters by inhibiting a specific cell-signalling pathway to improve the effect of chemotherapy and reverse tumour drug resistance in gemcitabine-resistant cells in pancreatic cancer, as demonstrated by QUI et al. [84].

### 4.5. Sonodynamic Therapy

Sonodynamic therapy (SDT) is a new therapeutic tool for non-invasive cancer treatment founded on the combined use of ultrasound and sonosensitizer drugs. Unlike conventional therapies, it is minimally invasive, site-specific, highly effective with minimal adverse consequences, and it is also capable of eliciting an antitumour immune response [126]. SDT produces reactive oxygen species (ROS) by ultrasonic excitation to kill cancer cells. Thanks to the creation of enough ROS, a cascade of biological events can be activated, including DNA fragmentation, cytoskeletal shrinkage, and chromatin condensation, leading to apoptosis [127]. It has been reported that sonoporation was successfully utilized to enhance nucleic acid delivery to neoplasms, skeletal muscle, and kidneys [128]. Sonoporation can be used with satisfactory results as a monotherapy or as an additional treatment option for inoperable or borderline resectable pancreatic ductal adenocarcinoma, as demonstrated in a study on ectopic murine pancreatic tumours [85]. For realizing minimally invasive cancer treatment, one study demonstrated the effectiveness and feasibility of drug delivery system-based SDT, which combined a small dose of NC-6300 and the low energy of HIFU in mouse models of pancreatic cancer [69]. Some new works have revealed good results by combining chemotherapy and sonodynamic therapy for the treatment of pancreatic tumours [58,86,87], while Nesbitt H et al. demonstrated that, when SDT was combined with anti-PD-L1 immune checkpoint inhibition in a murine model of pancreatic cancer, it induced a significant decrease in tumour volume when compared to treatment with SDT only [88]. Pigula M et al. showed the unique ability of longitudinal treatment monitoring to reveal a tumour size-dependent response to benzoporphyrin-derivative photodynamic therapy and irinotecan [89].

### 4.6. Harmonic Motion Elastography (HME) and Shear Wave Elastography (SWE)

Stiffness is an important biomechanical property of tumours. Harmonic motion imaging (HMI) is a radiation-force-based ultrasound elasticity imaging technique that is used for both tissue-related stiffness imaging and high-intensity focused ultrasound (HIFU) treatment monitoring [129]. Radiofrequency signal tracking is the method on which HMI was based to localize oscillatory motion caused by the harmonic radiation force given by two focused ultrasound transducer elements with overlapping beams oscillating at distinct frequencies [130]. Studies have indicated that using electronic steering can greatly increase the rate of tissue coagulation and reduce the total treatment time [129,131]. In this view, the harmonic motion imaging system can help as a new clinical tool for HIFU ablation monitoring [67,68].

Ultrasound elastography can measure tissue stiffness noninvasively and can be performed during theroutine imaging of some tumours. Among the techniques used to acquire elastographic data, shear-wave elastography (SWE) allows for more reproducible, quantitative measurement of tissue stiffness and yields quantitative SWE parameters, such as the minimum, mean, and maximum elasticity in a region of interest [132]. In addition, elastography aims to quantitatively image the Young’s E modulus, the physical parameter corresponding to the stiffness. It exhibits important variations between different biological tissues, which makes it ideal for the characterization of different tissues with an excellent contrast. Young’s modulus characterizes the stiffness of a tissue, which is exactly the quantitative reproduction of a clinician’s palpation and has relevant diagnostic value [133].

Therefore, harmonic motion elastography (HME) is a quantitative ultrasound-based imaging method used to calculate Young’s modulus (YM) in human and mouse models of pancreatic adenocarcinoma. HME has been used to estimate pancreatic rigidity in the murine representation of pancreatitis and pancreatic cancer and in several types of recently resected human pancreatic tumours [90]. It also has the ability of differentiating between different levels of fibrosis in transgenic mice based on the change in collagen density for detecting, staging, and delineating PDAC tumour margins [91,92]. In addition, by using STL-SWE (single-track location-shear wave elastography), Ahmed R et al. demonstrated, for the first time, that the stiffness changes occurring inside metastatic murine pancreatic tumours, as in liver metastasis, can be monitored over long-time scales (up to 9 weeks) [93]. Alvarez R et al. used endoscopic ultrasound elastography to evaluate the effects of nab-paclitaxel and gemcitabine in a mouse model of advanced pancreatic cancer, assessing tumour softening [94]. In a recent study, to characterize the performance of two newly optimized ultrasound-based analyses, shear wave and H-scan scattering analyses were applied to repeated trans-abdominal ultrasound scans of a murine model of metastatic pancreatic cancer [95].

### 4.7. Ultrasound-Guided Procedures

Ultrasound-guided intervention radiology procedures are a popular and valuable tool for performing imaging-guided procedures, providing good quality and real-time visibility of the needle or of the instrument to be advanced in the subject tissues.

In order to design new drugs to counter pancreatic adenocarcinoma, it is necessary to develop preclinical mouse models that best reproduce in vivo characteristics of this tumour. For this purpose, it is possible to use the ultrasound-guided injection of human pancreatic cancer cells to create an orthotopic xenograft mouse model of pancreatic cancer, as demonstrated by some recent studies [96,97,98]. Huynh AS et al. compared ultrasound-guided injection to highly invasive surgical orthotopic injection methods [99]. Surgical orthotopic tumour implantation models of PDA maintain the immunobiological hallmarks of the specific tumour microenvironment (TME) but require a time-intensive procedure and introduce aberrant inflammation. In another study, pancreatic cancer was induced in an ultrasound-guided orthotopic mouse model to evaluate the effects of high-affinity peptides on reversing chemotherapy-induced multidrug resistance (MDR) [100]. Through ultrasound guidance, pancreatic tumour cells were implanted orthotopically and led to pancreatic tumour formation in mice to analyse tumour-suppressive effects by treatment with a synthetic lipopeptide or intratumoral immunization with tumour RNA-pulsed dendritic cells [101,102].

Other studies have assessed the therapeutic efficacy of endoscopic ultrasound (EUS)-guided injection of ethanol versus 3-bromopyruvate to treat the orthotopic xenograft murine model of pancreatic cancer obtaining tumour necrosis, and the procedure was safe and effective [103,104].

## 5. Conclusions

In conclusion, through our research, it was seen that ultrasound can be a really useful, eclectic, and ductile tool in different diagnostic areas, not only regarding diagnosis but also in therapy, pharmacological and interventional treatment, and follow-up. All these multiple possibilities of use certainly represent a good starting point for the effective and wide use of murine ultrasonography in the study and comprehensive evaluation of pancreatic cancer with real translational perspectives.

## Data Availability

No new data were created or analysed in this study. Data sharing is not applicable to this article.
